# Potential of ILRIS3D Intensity Data for Planar Surfaces Segmentation

**DOI:** 10.3390/s90705770

**Published:** 2009-07-20

**Authors:** Chi-Kuei Wang, Yao-Yu Lu

**Affiliations:** Department of Geomatics, National Cheng Kung University, 1 University Road, Tainan 701, Taiwan; E-Mail: alcazer@gmail.com

**Keywords:** LIDAR, point cloud, laser scanning, ILRIS3D, lidar intensity

## Abstract

Intensity value based point cloud segmentation has received less attention because the intensity value of the terrestrial laser scanner is usually altered by receiving optics/hardware or the internal propriety software, which is unavailable to the end user. We offer a solution by assuming the terrestrial laser scanners are stable and the behavior of the intensity value can be characterized. Then, it is possible to use the intensity value for segmentation by observing its behavior, i.e., intensity value variation, pattern and presence of location of intensity values, etc. In this study, experiment results for characterizing the intensity data of planar surfaces collected by ILRIS3D, a terrestrial laser scanner, are reported. Two intensity formats, grey and raw, are employed by ILRIS3D. It is found from the experiment results that the grey intensity has less variation; hence it is preferable for point cloud segmentation. A warm-up time of approximate 1.5 hours is suggested for more stable intensity data. A segmentation method based on the visual cues of the intensity images sequence, which contains consecutive intensity images, is proposed in order to segment the 3D laser points of ILRIS3D. This method is unique to ILRIS3D data and does not require radiometric calibration.

## 1. Introduction

LIght Detection And Ranging (LIDAR) is an active remote sensing system that uses a pulse or continuous-wave laser to gather 3D information of the terrains and buildings at day or night [1]. The LIDAR point cloud can further be used for 3D building modeling [2], vegetation classification [3–5], etc. Many of the algorithms rely solely on the geometric properties of the remote sensed objects represented by the LIDAR point cloud [2,3]. Others employ the LIDAR intensity to refine data processing workflow [4–6].

For TLS (terrestrial laser scanner), or ground-based LIDAR, the intensity is usually recorded as an extra variable in addition to the 3D position information. The LIDAR intensity is a function of the reflectance and the texture of the target surface, the distance between the laser and the target, the angle of incidence of the laser beam impinging on the target surface, the transmitted power of laser, and the atmosphere attenuation coefficient of the air through which the beam has traveled [4,6–16].

However, the dynamic range of the LIDAR system can also affect the intensity. For example, the FARO LS HE80, with a dynamic range of 9 bits (= 128 levels), employs a logarithmic amplifier for short distance (∼5 m), which results in an abrupt change of intensity [12]. A correction attempt on the intensity values of the FARO LS HE80 was conducted by considering the aperture size effect of the detector and laser optics [16]. This implies the correction of the intensity should be made before the point cloud segmentation. However, this may require the knowledge of the proprietary internal process of LIDAR system, which is usually unavailable to the user, as is the case for ILRIS3D examined in this study [17]. On the other hand, airborne LIDAR systems usually have a greater dynamic range (> 11 bits) to account for different scenarios, and the inversion algorithms that have been developed by [7,9] are less concerned about this constraint and showing promising results.

Thus far, the intensity information contained in data collected by TLS has received much less attention compared to the geometric characteristic within TLS data ([18,19] and references therein). This is mostly due to the lack of information of converting the TLS intensity to more acquainted quantity, such as reflectance, which hampers the interpretation of the intensity information and the use of it for classification or segmentation purpose. In this paper, we believe that we are the first to offer a solution to conduct the segmentation task solely based on intensity within TLS data. The underlying assumption is that the terrestrial laser scanners are stable and the behavior of the intensity value can be characterized, which is confirmed by the experimental results from the FARO LS HE80 [12,16]. Similar characteristics are expected for other LIDAR systems, since all of them are designed and manufactured based on similar technology. Then, it is possible to use the intensity value for segmentation by observing its behavior, i.e. intensity value variation, pattern and presence of location of intensity values, etc. In this study, we conducted a series of experiments to characterize the intensity of ILRIS3D, a TLS with a dynamic range of 8 bits, of planar surfaces. A segmentation method will be proposed following the experiment results. This method will be empirical and can be regarded as a data driven approach [9].

## 2. Methods and Material

### 2.1. ILRIS3D

The pulse laser of ILRIS3D emits the wavelength of 1,535 nm with the beam divergence of 0.17 mrad [20]. The scanning mirror directs the laser beam ± 20 degrees in both vertical and horizontal directions, and it is suitable for targets within the range of 3–800 m. The unit is controlled by a Personal Digital Assistant (PDA) via an infrared communication port, and is powered by an external rechargeable battery with a nominal operation time of 5 hours. The ILRIS3D display screen shows the scan settings and scan progress information of the unit and the status of the battery that is connected to it.

The power of the return laser beam measured by ILRIS3D is in 16 bits. ILRIS3D outputs two intensity formats, grey (*I_G_*) and raw (*I_R_*), each ranges 0–255 and 0–25500, respectively. Both of the formats are 8 bits. The one-to-one relations are:

(1) $$\begin{array}{c} I_{G}=127+I_{R}/200,\;\text{for low gain},\;\text{and}\\ I_{G}=I_{R}/2,\;\text{for high gain} \end{array}$$

An internal process is applied for scaling and conversion from 16 bits to 8 bits by the manufacturer design. Unfortunately, the detailed information concerning what kind of conversion is applied to the intensity is unavailable to the user [17].

### 2.2. Experiments

It is implied from Equation (1) that no difference exists between the two intensity formats, if the one-to-one conversion is applied. However, detailed examination of the data indicates otherwise (results not shown). Thus, a series of experiments is conducted to characterize ILRIS3D intensity and to determine which intensity format should be used for segmentation applications.

A normal use of ILRIS3D would last from several hours to few days, depending on the size and the complexity of the project, and one concern is that the transmitted laser pulse power is not constant from the start to the end of a scan operation due to ILRIS3D’s compact design. This temporal variation can be easily monitored and then accounted for by adding a channel to record a certain percentage of the transmitted laser [1,7,13]. However, most terrestrial LIDAR, such as ILRIS3D used in this study, lack this design feature.

Experiments on the temporal intensity variation are conducted in an indoor environment. A complete scan, starting from a fully charged battery until it runs out of power, includes repeated scans of a spectralon panel (size of 25.4 cm by 25.4 cm) of 75% reflectance at 7 m away from the ILRIS3D. The scan density is set at 5 mm and a single scan of the spectralon panel can be finished within 5 minutes. The mean and standard deviation of each single scan of all the intensities on the spectralon are calculated.

**Figure 1.**
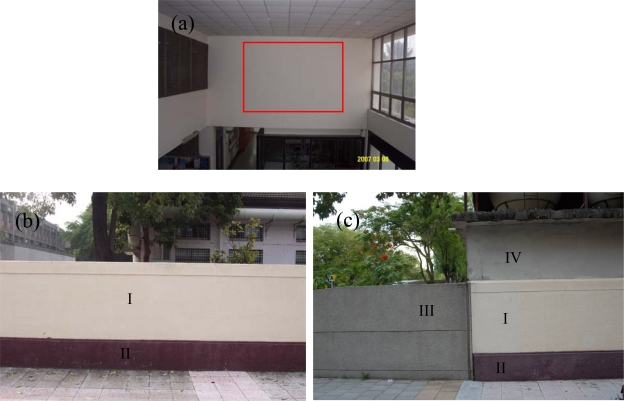
(a) The homogeneous white wall, with the red rectangle denoting the scan area. (b) The flat concrete fence of yellow (surface I) and brown (surface II), (b) A scene including surfaces I – IV. Surface III is a small-grain decorated fence (dark grey); surface IV is a smooth concrete wall (light grey).

The first experiment case of the planar surfaces is a homogenous flat white wall 25 m away from the ILRIS3D [the red rectangle in Figure 1(a)]. The second is a concrete fence painted with two different colors (yellow and brown) 5 m away from ILRIS3D [Figure 1(b)]. The third is a scene including the concrete fence in the second experiment, a smooth concrete wall (light grey), and a small-grain-decorated fence (dark grey) [Figure 1(c)] 6 m away from ILRIS3D. All of the scans of the above mentioned outdoor surfaces are collected by one laser scan.

## 3. Results and Discussion

### 3.1. Temporal Intensity Variation

**Figure 2.**
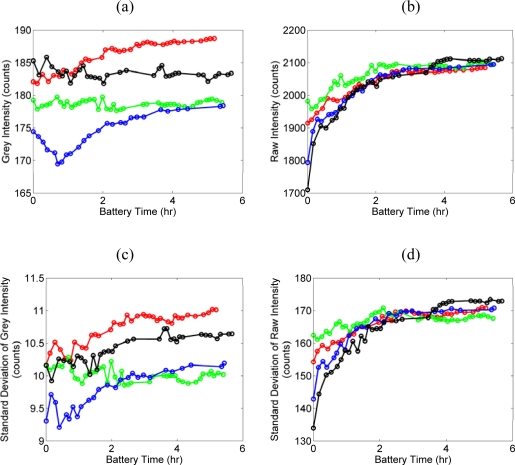
Time series of five complete scans of the battery experiment. (a) mean of grey intensity, (b) mean of raw intensity, (c) standard deviation of grey intensity, and (d) standard deviation of raw intensity.

Five complete scans of the 75% reflectance spectralon panel are shown in Figure 2, which indicates more than 5 hours of scan operation for a fully charged battery for each scan. The means of both the grey intensity [Figure 2(a)] and the raw intensity [Figure 2(b)] increases with increasing battery time. The intensity range, defined as the difference between the maximum and minimum mean intensity of one single complete scan, is less than 17.4 and 402.7 for the grey and the raw intensities, respectively. The standard deviation of the grey intensity [Figure 2(c)] and the raw intensity [Figure 2(d)] also increases with increasing battery time, and the variance of the grey intensity is significantly less than that of the raw intensity. When the standard deviation reaches its maximum (usually after 3 hours of battery time), the ratio of the standard deviation to the mean of the grey intensity is ∼0.06, and that of the raw intensity is ∼0.08. The smaller ratio value indicates a smaller intensity variation, which is more preferable for segmentation applications, and thus, the grey intensity should be used in this case.

Usually after approximately 1.5 hours of battery time, the ILRIS3D display screen indicates the battery is at “LOW” status. Considering only data collected when the battery is “LOW”, the intensity range of grey and raw intensities are reduced to less than 3.9 and 87.2, respectively. For the grey intensity, the variation is reduced to 22.4% (= 3.9/17.4 × 100%) of the original value, and this can be interpreted as a warm-up time increases the stability of ILRIS3D intensity.

The nominal warm-up time of ILRIS3D is 0.5 hours [17]. However, our experiment results show better stability of the grey and the raw intensity after 1.5 hours [Figure 2]. Unfortunately, this warm-up phenomenon cannot be modeled or predicted. A proprietary mechanism called ASC, Automated Scan Correction, is implemented by the manufacturer to compensate for temperature drift of range and angular measurements, but not for intensity [17]. The access to the information regarding ASC is restricted by the manufacturer. We are unable to assess whether ASC is useful for intensity related use, i.e. predicting the warm-up phenomena of the intensity. Consequently, all the data collected afterwards are at battery “LOW” status to obtain consistent LIDAR intensity.

### 3.2. Intensity Image and Intensity Image Sequence

**Figure 3.**
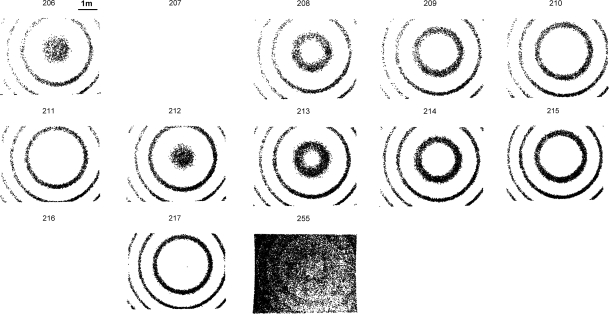
Grey intensity images of 206 to 217 and 255 excerpted from the grey intensity image sequence of a homogeneous white wall [red rectangle in Figure 1(a)] in Supplementary Material 1, which contains the complete grey intensity of 199–255.

The ILRIS3D are setup approximately parallel to the normal vector of the planar surfaces. The intensity image is created for each intensity value, and each intensity image contains lidar point cloud of one single intensity value of the same viewing perspective as the terrestrial scanner gathers the 3D points. The intensity image sequence is then created by composing the intensity images into one single file, with increasing intensity value order (no temporal information is used). Any software with 3D graphic capability for displaying point data can be used for the creation of the intensity image. In this study, we created the intensity image in Matlab (MathWorks, Inc.). Figure 3 shows the grey intensity images of 206–217 and 255 from the results of the white surface experiment [the surface is subtended by the red rectangle in Figure 1(a)], while the intensity image sequence in Supplementary Material 1 shows the complete intensity range (no grey intensity below 199). Figure 3 is also a clear evidence that the angle effect of ILRIS3D grey intensity is significantly different than that reported by others, where the intensity is monotonically decreasing with increasing angel of incidence [4,9,11,13,14]. The grey intensity of 255 is saturated and provides no information for segmentation. This is due to the internal process of intensity scaling and conversion from 16 bits to 8 bits [17].

### 3.3. Planar Surface Scans

For the white surface experiment [red rectangle in Figure 1(a)], the concentric circular pattern, is prominent in the intensity image sequence from 200 to 253, shown in Supplementary Material 1, with a repeating frequency of every 5 or 6 grey intensities. Intensity images of 206–217 and 255 are excerpted from the intensity image sequence for illustration and are shown in Figure 3. For this experiment, except for the empty intensity image, such as 207 and 216 shown in Figure 3, where on lidar point cloud is collected at this intensity value, there are approximately 4 concentric circles in each intensity image. The intensity images of 206–211 show a complete cycle of the evolving pattern for a planar surface obtained by ILRIS3D, where the radii of the circles are increasing with increasing grey intensity values, while the most inner circle starts (grey intensity of 206) as a fuzzy circle encompassing a large amount of lidar points. Another cycle of the evolving pattern can be seen with the intensity images from 212 to 217 in Figure 3. The intensity image sequence in Supplementary Material 1 shows a total of 9 cycles of evolving pattern of the concentric circles for the white surface experiment, where the starting intensity images are 200, 206, 212, 218, 223, 229, 234, 241, and 247, respectively. Although the evolving pattern of the concentric circles is different in details for the 9 cycles (Supplementary Material 1), they are consistent in general appearance and can be easily identified. Given the grey intensity format (of 8 bit dynamic range) employed by ILRIS3D, the saturated signals are expected to be contained in the intensity image of 255, shown in Figure 3, where lidar points are scattered all over the white planar surface, and doesn’t provide distinctive visual cues as those of the concentric patters in intensity images of 206–253. This result from the white surface experiment suggest that the visual cue of the varying radii can be used a criteria to segment ILRIS3D data into a planar surface.

**Figure 4.**
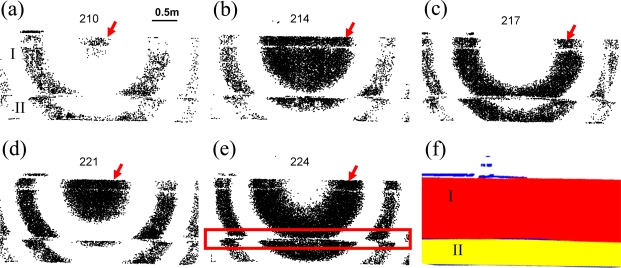
A concrete fence painted with two colors [cf. Figure 1(b)]: (a)–(e) Grey intensity image of 210, 214, 217, 221, and 224, excerpted from the intensity image sequence in Supplementary Material 2, which contains the grey intensity from 187 to 255. The red rectangle denotes the linear discontinuity feature between the concentric circular patterns of surface I and II. See the text in Section 3.3 for the explanation of the red arrows. (f) Segmentation result using the visual cues of two distinct concentric circular patterns and a linear feature [denoted by the red rectangle in (a)] in the intensity image sequence in Supplementary Material 2. Red and yellow denote surfaces I and II, respectively, while blue represents the un-segmented 3D laser points.

This concentric circular pattern is due to the internal process of intensity scaling and conversion from 16 bits to 8 bits by the manufacturer design [17]. The grey intensity images of 210–224, which represent a complete cycle of the evolving pattern, and the intensity image sequence of a concrete fence painted with two different colors [Figure 1(b)] are shown in Figure 4(a)–(e) and Supplementary Material 2, respectively. Because the fence is 5 m away from the ILRIS3D and the height of the wall is 1.8 m, only one-half of the concentric circular pattern with varying radii is visible for surface I; an even smaller portion of that is visible for surface II [Figure 4(a)–(e) and Supplementary Material 2]. For surface I, the concentric circular pattern is half circles and the centers of these half circles appear to be at the top edge of surface I, which can be seen in grey intensity images of 210, 227, and 244 shown in Supplementary Material 2. For surface II, only an arc-like feature can be recognized, and the centers of these arcs appear to be located slightly below the top edge of surface I. The repeating frequency is 17 grey intensities, which is different than that of the white planar surface. And, only one-third of the intensity images contain the 3D laser points, i.e. except for grey intensity of 187, 190, 193, etc, all intensity images are empty. The radii of the concentric circles, shown in Figure 4(a)–(e) and Supplementary Material 2, do not increase with increasing intensity values as the white surface experiment (Figure 3). For example, the evolving pattern of the most inner circle of surface I, denoted by the red arrows in Figure 4(a)–(e), experiences the following stages within a complete cycle: starts as a small fuzzy circle [Figure 4(a)], becomes a large circle [Figure 4(b)], evolves to a band-like feature [Figure 4(c)], turn back to a circle [Figure 4(d)], becomes a band-like feature with large width, again [Figure 4(e)]. The evolving pattern of the varying radii can be more easily identified with the use of intensity image sequence in Supplementary Material 2. The presence of ring- and arc-like features, change of repeating frequency, and the empty grey intensity images are all due to the internal process of intensity scaling and conversion from 16 bits to 8 bits [17].

Following the results implied by the white planar surface experiment (Figure 3), that the point clouds of the varying radii can be segmented into the same surface, we are able to confirm the existence of surfaces I and II by observing the intensity image sequence in Supplementary Material 2. A linear distinctive discontinuity feature can also be found at the boundary of the two surfaces [denoted as red rectangle in Figure 4(a)] due to different color paintings of the two surfaces, which substantiates segmenting the point cloud into two planar surfaces. A GUI (graphic user interface) program is developed in Matlab (MathWorks, Inc.), which facilitates the manual selection process of segmenting the lidar points into two surfaces, and the result is shown in Figure 4(f), where red and yellow denote surfaces I and II, respectively, while the un-segmented 3D laser points are denoted as blue.

Because the grey intensity range for surfaces I and II are 187–251 and 190–251, respectively, a simple threshold is insufficient to separate the point cloud of the two surfaces, even though they are visually different in color.

**Figure 5.**
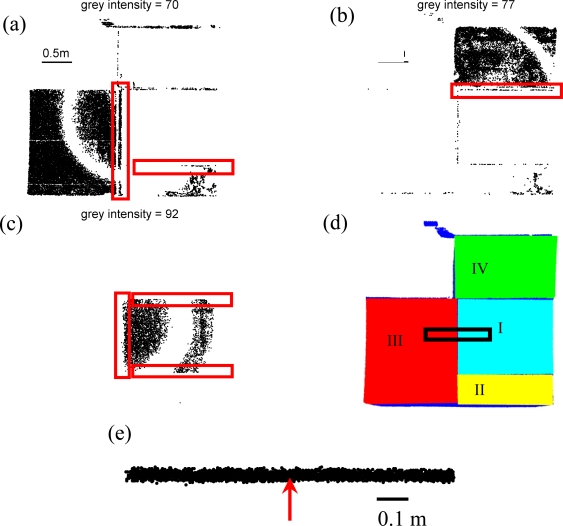
Results of four different surfaces [Figure 1(c)]: (a) grey intensity image of 70 showing surfaces III and II, (b) grey intensity image of 77 showing surface IV, (c) grey intensity image of 92 showing surface III, (d) Segmentation results using the visual cues of the concentric circular patterns and linear features dividing each concentric circular pattern [red rectangles in (a)–(c); see the text in Section 3.3 for explanation] shown in the intensity image sequence in Supplementary Material 3. Cyan, yellow, red, and green denote surfaces I, II, III, and IV, respectively, while blue represents un-segmented 3D laser points. (e) The nadir view of the subset of point cloud excerpted from the black rectangle in (d). The red arrow indicates the boundary between surface I and III in (d).

The results from the scan of the four planar surfaces [Figure 1(c)] are shown in Figure 5. The grey intensity ranges for surfaces I–IV, shown in Table 1, are smaller than those tested in previous experiments. Thus, the repeating frequency can not be determined for surfaces I–IV of this experiment. For surfaces I, III, and IV, the smallest height and width are 1 m and 1.5 m, respectively; the height and width of surface II is 0.5 m and 1.5 m, respectively. The size of each surface is smaller than those tested in previous experiments. And only a quarter or less of the circular pattern is visible [Figure 5(a)–(c) and intensity images sequence in Supplementary Material 3]. For this experiment, the boundaries separating different surfaces, denoted as red rectangles in Figure 5(a)–(c), can be distinctively identified, which is helpful in lidar point segmentation.

For surfaces I, III, and IV, the circular pattern is large enough to be recognized as different surfaces. Due to the small dimensions of surface II, the concentric circular pattern is not prominent. The identification of surface II can be facilitated by the presence of the boundaries, which are linear features and denoted as the red rectangles in Figure 5(a) and 5(c). The red rectangle shown in the middle of Figure 5(a) is a boundary separates two groups of varying radii which represent surfaces III and II, respectively. Due to limited number of grey intensity, the concentric circular pattern of surfaces I and II do not appear in the same intensity range (Table 1), and the other red rectangle shown in Figure 5(a) is the top boundary of surface II, where the same boundary (which becomes the bottom boundary of surface I) can be found as the bottom-right red rectangle in Figure 5(c). The other boundaries can also be found for surface IV in Figure 5(b), and surface I in Figure 5(c). Thus, in addition to the visual cues of the concentric circular pattern, the linear boundaries, which separates different group of varying radii or delineates the boundary of a group of vary radii, are helpful for the segmentation task.

Figure 5(d) shows the segmentation results using the visual cues of the concentric circular patterns of these small surfaces and boundaries, appearing as linear features dividing each concentric circular pattern shown in the intensity image sequence in Supplementary Material 3, using the GUI program by manual selection. The order of which grey intensity value is processed is of no importance because they will be distinctively different groups of lidar points of varying radii in the intensity image sequence.

The information of displacement and variation in the point cloud data is usually employed by geometric based segmentation algorithm [2,3,18,19]. Figure 5(e) shows the nadir view of a subset of point cloud extracted from the black rectangle in Figure 5(d), and the red arrow indicates the boundary between surfaces I and III. It is evident in Figure 5(e) that the variation and displace of point clouds for the two surfaces are similar, which implies a geometric based segmentation algorithm is expected to produce non-satisfactory results because the information of intensity, related to the color and texture, is not considered.

Table 1 shows the grey intensity range for surfaces I–IV. Some of the intensity ranges are overlapping, for example, surfaces II and III, which preclude the use of a single threshold to segment the grey intensity.

For the three experiments conducted in this study, the surfaces are 25 m, 5 m, and 6 m away from the ILRIS3D (Figure 1). All of the experiment results show similar patterns of concentric circles and distinct linear features at the boundaries of two surfaces (Figure 3–5). It is expected that this pattern of the intensity data can be found in other dataset of surfaces collected by ILRIS3D at greater distance.

## 4. Conclusions

Due to the limited dynamic range of ILRIS3D, point cloud segmentation algorithms based on the radiometric information [1,4,6,7,9,10,12] are not applicable. Grey intensity format is found to be more consistent among the two intensity formats employed by ILRIS3D. A warm-up time of 1.5 hour is suggested, if permitted in a scan operation, in order to achieve higher consistency in grey intensity. This warm-up time is indeed too long, and may result in a prolonged scan operation. This implies that ILRIS3D is not yet optimized for intensity measurement. We suggest a new mechanism for compensating the temperature variation of intensity in the future units by the manufacturer in order to exploit the information contained within the intensity.

We propose a new approach by the use of the intensity images sequence, which consists of consecutive intensity images, for point cloud segmentation of ILRIS3D datasets. The procedures are as follows:

Step 1. Construct gray intensity images. Gray intensity images with no point cloud can be discarded if desirable.
Step 2. Construct intensity image sequence by composing the grain intensity images in sequence.
Step 3. Identify the presence of surfaces by recognizing the visual cues of the evolving pattern of concentric circle with varying radii in the intensity image sequence.
Step 4. Identify the presence of boundaries by recognizing the linear features that separate different group of varying radii or delineate the boundary of a group of vary radii.
Step 5. Group the point cloud into different planar surfaces according to the visual cures of the concentric circular pattern in Step 3 and linear features identified in Step 4 by manual selection.

Although our results are preliminary, our method provides a working solution for the segmentation of 8 bit terrestrial laser scanner data. In addition, our method considers the color and texture information embedded in the lidar intensity, which is often not used by geometric based algorithms (Figure 5) [2,3,18,19]. According to the experiment results from FARO HE80, a 9 bit system, the alteration of intensity data is evident and the use of that information is not trivial [12]. Our method may provide a solution to that and possibly to other system storing intensity in 8 or 9 bits formats [15]. For other lidar systems with greater dynamic ranges, the inversion algorithms [7,9] should be applicable.

The concentric patterns are similar in the three cases presented in this article [Figure 3–5 and Supplementary Material 1–3], but they are different in detail. Hence, we suggest the point cloud segmentation using intensity image sequence should be conducted by the operator to ensure high correction rate. Before a more comprehensive result for characterizing the intensity data of ILRIS3D is available, which should include the input from the manufacturer regarding the internal data processing by the onboard hardware, we do not recommend an automatic procedure for our method due to variations in the details in the image patterns.

The high point cloud density of 5 mm, as was chosen in this study, is not required. The point cloud density should be determined according to the feature of interest in the scan scene. The segmentation result using our method can be produced with minimal computation time, because there is no computational effort needed to determine the displacement and variation information within the point cloud [18,19], which enables a quick assessment of point cloud data on site.

## Figures and Tables

**Table 1.** Grey intensity ranges for surfaces shown in Figure 1(c).

**surfaces**	**I**	**II**	**III**	**IV**
grey intensity range (counts)	82–99	59–80	66–75	73–85
